# Effect of up‐regulation of circMATR3 on the proliferation, metastasis, progression and survival of hypopharyngeal carcinoma

**DOI:** 10.1111/jcmm.15134

**Published:** 2020-03-12

**Authors:** Zhanwang Wang, Peng Wei, Dongmin Wei, Shengda Cao, Heng Liu, Long Chen, Xiao Han, Xiaoyan Zhao, Chuan Liu, Guojun Li, Jianming Yang, Xinliang Pan, Dapeng Lei

**Affiliations:** ^1^ Department of Otorhinolaryngology Qilu Hospital Shandong University Key Laboratory of Otolaryngology NHFPC (Shandong University) Shandong China; ^2^ Department of Biostatistics The University of Texas MD Anderson Cancer Center Houston TX USA; ^3^ Department of Otorhinolaryngology The First Affiliated Hospital of Chongqing Medical University Chongqing China; ^4^ Department of Head and Neck Surgery The University of Texas MD Anderson Cancer Center Houston TX USA; ^5^ Department of Otolaryngology Head and Neck Surgery The Second Hospital of Anhui Medical University Hefei China

**Keywords:** circMATR3, circular RNAs, hypopharyngeal squamous cell carcinoma, progression

## Abstract

Increasing number of circular RNAs (circRNAs) have been reported to play important role in gene regulation, carcinogenesis and pathogenesis in various cancers. However, the biological functions and underlying molecular mechanisms of circRNAs in hypopharyngeal squamous cell carcinoma (HSCC) remain elusive. Thus, secondary circRNA‐seq profiling was performed to identify the differentially expressed circRNAs between HSCC tissues and adjacent normal tissues, and the expression level of circMATR3 (derived from human gene matrin3 (MATR3), has_circRNA_0008922) was confirmed by qRT‐PCR. Proliferation of HSCC cells was detected by cell counting kit‐8 (CCK8) assay, apoptosis and the cell cycle were analysed by flow cytometry, and the migration and invasion of HSCC cells was determined by transwell assay. Bioinformatics analysis was conducted to predict possible pathways and potential miRNA targets of circMATR3. We found that circMATR3 was up‐regulated in HSCC tissues, and abundant circMATR3 expression was markedly correlated with late T classification, advanced clinical stage, greater lymph node metastasis, and poor prognosis. Furthermore, knock‐down of circMATR3 significantly inhibited proliferation, migration and invasion of HSCC cells, whereas silencing of circMATR3 induced cell apoptosis. Our analysis predicted that circMATR3 may participate in cancer‐related pathways by serving as miRNA sponges. In conclusion, our findings first identified the oncogenic roles of circMATR3 in promoting the progression of HSCC and demonstrated that circMATR3 may be a novel prognostic marker and therapeutic target for HSCC.

## 1 INTRODUCTION

Hypopharyngeal squamous cell carcinoma (HSCC) is an aggressive tumour arising from the outer layer (epithelium) of the upper aerodigestive tract^1^ and accounts for 5%‐15% of all head and neck squamous cell carcinoma (HNSCC).^2^ Due to the recent improvements in modern comprehensive treatment strategies including surgery, chemotherapy and radiotherapy, the 5‐year survival rate for patients with early‐stage HSCC is as high as 70%^3^; however, the majority of HSCCs are diagnosed at an advanced stage (stage III or IV), with a 5‐year survival rate of about 35%,^4^, ^5^ and HSCC patients are also vulnerable to relapse. Therefore, it is critical to identify new prognostic biomarkers and therapeutic targets facilitating the early diagnosis of HSCCs and improving the clinical outcomes of patients.

Circular RNAs (circRNAs) are a novel class of non‐coding RNAs that are characterized by covalently closed‐loop structures, without a polyadenylated tail and 5′ to 3′ polarity that endow the conserved and stable structures for circRNAs,^6^ which enables circRNAs to resist to the degradation by RNA exonuclease or Ribonuclease R (RNase R).^7^ Originally, circRNAs were misinterpreted as the by‐products of splicing errors when they had been occasionally identified from several transcribed genes over two decades ago.^8^ However, with the development of novel bioinformatics analysis and high‐throughput sequencing technology, numerous circRNAs have been identified in different species and various mammals cell lines. Furthermore, emerging evidence shows that circRNAs may play critical roles in many pathophysiological and physiological processes by acting as microRNA sponges, regulators of splicing and transcription, RNA binding proteins (RBPs) to sequester miRNAs^9^, ^10^ and regulate transcription processes or alternative splicing^11^ as well as gene expression.^12^ Recently, increasing studies have suggested that circRNAs regulate the proliferation, invasion, migration, apoptosis and therapeutic resistance of cancer cells in various kinds of human cancers,^13^ including hepatocellular carcinoma,^14^ gastric cancer,^15^ colorectal cancer^16^ and bladder cancer.^17^ These reports indicate that circRNAs may be emerging prognostic biomarkers and therapeutic targets in human cancers. In our previous study,^18^ we identified the different expression of circRNAs between HSCC tissues and adjacent normal tissues via microarray analysis. However, due to the limitation of microarray analysis, a large number of novel circRNAs beyond previous microarray analysis have yet to be identified. Moreover, the functions, clinicopathological significance and molecular mechanisms of circRNAs in HSCC have never been investigated.

Matrin3 (MATR3) is a highly conserved human gene, which is known to stabilize certain messenger RNA species through coding a nuclear matrix protein. Mutations of this gene are closely associated with vocal cord and pharyngeal weakness. MATR3 is also involved in chromatin remodelling, DNA replication, RNA processing, transcription, translation and apoptosis.

In this study, we first performed circular RNA sequencing profiling analysis and identified that circMATR3 (derived from exon3 and exon4 of MATR3 gene, hsa_ circ_0008922) is significantly up‐regulated in HSCC samples and closely associated with poor clinicopathological features and worse prognosis for patients with HSCC. In vitro experiments demonstrated that circMATR3 plays crucial roles in cell proliferation, apoptosis, migration and invasion of HSCC cells. Furthermore, we predicted that circMATR3 may exert its oncogenic role in HSCC by serving as miRNA sponges and regulating cancer‐related pathways. Therefore, increased expression of circMATR3 may act as a novel prognostic marker and therapeutic target for HSCC patients.

## 2 MATERIALS AND METHODS

### 2.1 Patients and specimens

Hypopharyngeal squamous cell carcinoma tissues and paired normal tissues were obtained from 55 HSCC patients who underwent surgery from January 2011 to January 2014 at the Qilu Hospital (Jinan, Shandong, China). In all patients, the diagnosis of HSCC was confirmed by pathological analysis and no patients underwent radiation therapy or chemotherapy before surgery, and all these patients were chosen randomly and underwent hypopharyngeal carcinoma resection plus ipsilateral modified neck dissection surgery. Five pairs of HSCC tissue samples were randomly selected for circular RNA‐seq profiling analysis, and another 50 pairs of HSCC specimens were used to confirm the circRNAs expression by quantitative real‐time polymerase chain reaction (qRT‐PCR). The clinical information of patients was collected from the patient history in the medical records. The HSCC was staged in accordance with American Joint Committee on Cancer TNM staging system.^19^ Patients returned for follow‐up visits once every 3 months until January 2019; and 5 patients were lost to follow‐up. All patients provided written informed consent for the study, and the consent procedure was approved by the institutional review board of the Ethics Committee of Qilu Hospital.

### 2.2 CircRNA‐seq profiling analysis

Total RNAs were extracted from fresh tissue samples by using TRIzol (Invitrogen) reagent in accordance with the manufacturer's instructions. The concentration and quality of total RNAs were determined with the NanoDrop ND‐2000 (Thermo Fisher Scientific), and the OD260/OD280 ratios of RNA range from 1.8 to 2.1 were deemed acceptable. Moreover, the RNA integrity was assessed by electrophoresis on a 1% denaturing agarose gel.

Total RNAs (5 μg) were pre‐treated to enrich circRNAs and remove the linear RNA by using CircRNA Enrichment Kit (Cloud‐seq Inc). RNA libraries were constructed by using pre‐treated RNAs with TruSeq Stranded Total RNA Library Prep Kit (Illumina) in accordance with the manufacturer's instructions. Libraries were controlled for quality and quantified using the BioAnalyzer 2100 system (Agilent Technologies, Inc). The RNA libraries were denatured as single‐stranded DNA molecules, captured on flow cells (Illumina), and amplified in situ as clusters and finally sequenced for 150 cycles on HiSeq 4000 Sequencer (Illumina) as described.^20^

### 2.3 qRT‐PCR

Total RNAs were extracted from tissues or cells by using TRIzol regent in accordance with the manufacturer's instructions. The cytoplasmic and nuclear RNA were extracted by using the PARIS™ Kit (Invitrogen AM1921). Approximately 1 μg of RNA from tissues or cells was subjected to cDNA synthesis using the reverse transcription kit (Takara RR047A), and qRT‐PCR was performed with use of a SYBR Green Premix Ex Taq kit (Takara RR820A). Relative quantification was assessed with use of an ABI 7900HT qRT‐PCR system using the 2^−ΔΔCt^ method, and 18s rRNA was used as the endogenous control. The sequences of primers are summarized in Table S1.

### 2.4 Ribonuclease R digestion

RNase R digestion reaction was conducted as previously described.^21^ Total RNA (2 μg) was incubated at 37°C for 30 minutes with 4 U/μg of RNase R (Epicentre, Inc) to enrich circRNAs, and after digestion, the total RNA was incubated at −80°C overnight to stop the reaction, and then, the RNA was subjected to cDNA synthesis and PCR. The amplification products were assessed by agarose gel electrophoresis.

### 2.5 Sanger sequencing

To confirm the back‐splice junction site of circMATR3, we designed divergent primers for performing PCR. The full length of PCR amplification products was determined by inserting them into a T‐vector for the Sanger sequencing. The primer synthesis and Sanger sequencing were performed by the Biosune Company.

### 2.6 Cell culture

The only human HSCC cell line in this study, FaDu cell line, was obtained from the American Type Culture Collection (ATCC). FaDu cell was maintained in Eagle's Minimum Essential Medium (EMEM; Gibco) supplemented with 10% foetal bovine serum (FBS; Gibco) and 1% Penicillin‐Streptomycin Liquid (Solarbio) in a humidified atmosphere with 5% CO_2_ at 37°C.

### 2.7 siRNA and cell transfection

The small interfering RNA (siRNA) targeting splicing junction site of circMATR3 (s1‐circMATR3 and s2‐circMATR3) and scrambled negative control (NC) were designed and synthesized by GenePharma. FaDu cells were seeded in 6‐, 12‐ and 96‐well plates, and after seeding 24 hours, they were transfected with a mixture of s1‐circMATR3 or s2‐circMATR3 (30 nmol/L, diluted in opti‐MEM medium) or a scrambled negative control (NC) and Lipofectamine 3000 (Invitrogen) in accordance with the manufacturer's protocols. Forty‐eight hours after transfection, qRT‐PCR was used to quantify the silencing efficiency of circMATR3 and MATR3 mRNA. The sequences of NC, s1‐circMATR3 and s2‐circMATR3 are shown in Table S1.

### 2.8 Cell counting kit‐8 assay

Proliferation of FaDu cells was detected using the CCK8 assay kit (Doindo). Approximately 7 × 10^3^ transfected cells in 100 μL were maintained in triplicate in 96‐well plates. The CCK8 reagent (10 μL) was added to every well, and then, the cells were incubated in a 5% CO_2_ atmosphere at 37°C for 2 hours. The optical density (OD) value at 450 nm was measured.

### 2.9 Transwell assays

We used transwell chambers (Corning) coated with or without Matrigel Matrix (Corning) to assess the invasion and migration of FaDu cells. After 24 hours of transfection, FaDu cells were collected with full trypsinization and then diluted to serum‐free EMEM with a density of 6 × 10^5^ cells/mL. A 200 μL cell suspension was plated into the upper chamber. EMEM (600 μL) containing 20% FBS was added to the lower transwell chamber, and the cells were incubated at 37°C for 48 hours. Cells had invaded to the lower chamber were fixed with methanol for 30 minutes and then stained with 0.1% Crystal Violet Stain solution (Solarbio) for 30 minutes. We utilized an inverted microscope (Olympus) to photograph the stained cells and count the stained cells manually.

### 2.10 Analysis of apoptosis via flow cytometry

Apoptosis of cells was determined using a FITC Annexin V Apoptosis Detection Kit (BD Biosciences 556547). FaDu cells were seeded into 6‐well plates at a concentration of 1.5 × 10^5^ cells/mL. After transfection with the two different siRNAs for 48 hours, cells were fully digested with EDTA‐free trypsin for 2 minutes and then centrifuged at 800 rpm for 5 minutes, and the supernatant was discarded. Cell pellets were then washed twice with ice‐cold phosphate buffer saline (PBS) and stained with 5 µL of FITC Annexin V and 5 µL of propidium iodide (PI) for 15 minutes at room temperature in the dark. The cells were added to 400 µL of binding buffer and observed by a flow cytometry. Results were analysed by using CytEpert v2.0 (Beckman Coulter, Inc).

### 2.11 Analysis of cell cycle

For cell cycle analysis, after transfection with the two different siRNAs for 48 hours, FaDu cells were fully digested and washed three times with ice‐cold PBS and fixed with 75% ice‐cold ethanol at −20°C overnight. The cells were centrifuged at 1000 × *g* for 10 minutes and then were washed with ice‐cold PBS and centrifuged at 500 × *g* for twice. After discarding the supernatant, we resuspended the cells with 0.5 mL PI/RNase Staining Buffer (BD Biosciences 550825) and incubated the cells for 15 minutes at room temperature in the dark, and then the cell cycle was immediately detected via a flow cytometer (Beckman Coulter, Cytoflex S) and analysed with use of Modfit software.

### 2.12 Prediction of circRNA‐miRNA‐mRNA networks and bioinformatics analysis

To explore the underlying mechanisms of circMATR3, we predicted the potential miRNA binding sites of circMATR3 by using Arraystar's homemade software based on miRanda and TargetScan. We constructed circRNA/miRNA/mRNA networks of five highest‐ranking miRNAs matched circMATR3 with the help of Cytoscape software (v3.7). In addition, we performed Gene Ontology (GO) and Kyoto Encyclopedia of Genes and Genomes (KEGG) pathway enrichment analysis for target mRNAs through the online website DAVID (v6.8).

### 2.13 Statistical analysis

All data were presented as the mean ± standard deviations (SD), and all experiments were independently repeated at least three times. Data were analysed using GraphPad Prism 6 software (San Diego Inc, CA, USA). One‐way ANOVA test was performed to analyse the difference between three experimental groups. Brown‐Forsythe test was used to check the variances of data. The Student's *t* test was performed to evaluate the difference of two groups, and the Shapiro‐Wilk test was used to check the normality of data. The relationship between circMATR3 expression and clinical features was analysed by using the Fisher exact test or chi‐square test. Kaplan‐Meier method was used to draw survival curves for HSCC patients with low or high circMATR3 expression. A *P* value < .05 (2‐sided) was considered as statistically significant.

## 3 RESULTS

### 3.1 RNA‐seq profiling of circRNAs in HSCC tumorous and paired adjacent normal tissues

**Figure 1:**
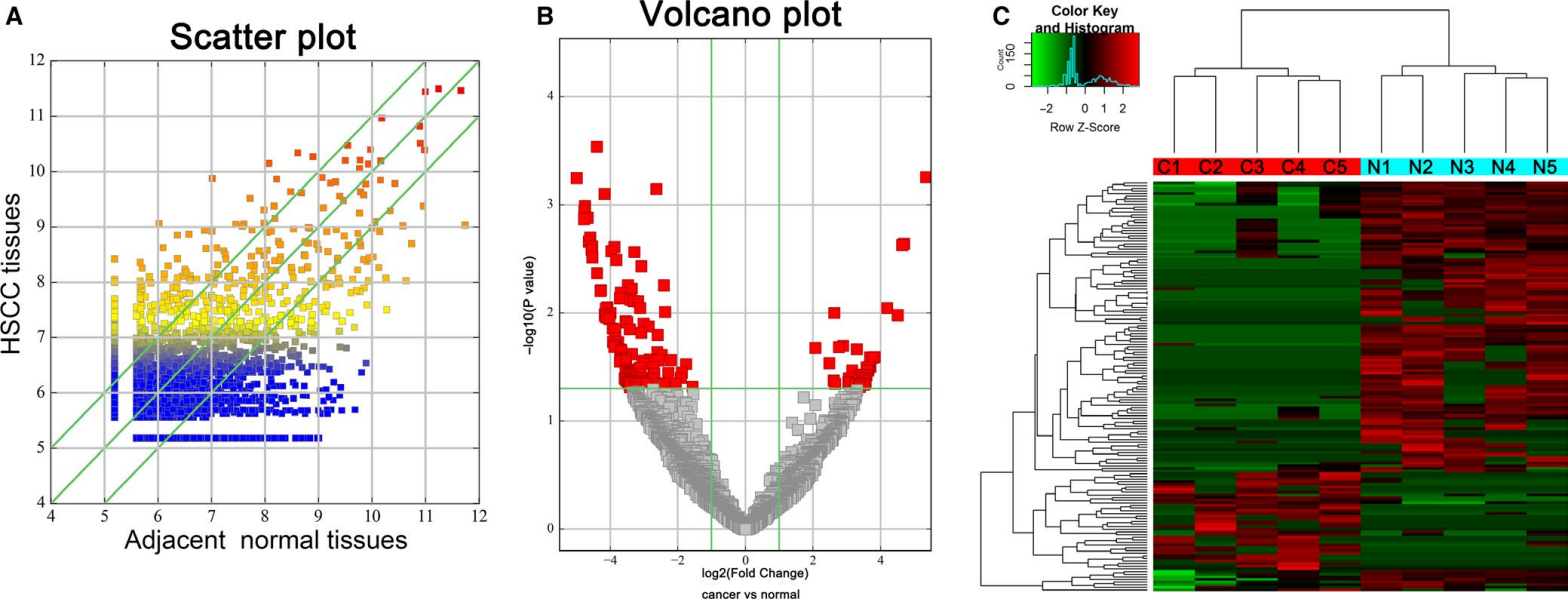
Profiles of differentially expressed circRNAs between HSCC tissues and paired adjacent normal tissues. A, The scatter plot shows the circRNA expression difference between HSCC samples and paired normal samples (the points above the topmost green line represent up‐expressed circRNAs and the points beneath the bottommost green line represent down‐expressed circRNAs with fold change ≥2.0, respectively). B, The volcano plot reveals differentially expressed circRNA in HSCC tissues (red frame represents markedly dysregulated circRNAs with fold change ≥2.0 and *P* value < .05). C, The clustered heatmap exhibits the differentially expressed circRNAs between 5 pairs of HSCC cancerous tissues (C1, C2, C3, C4 and C5) and adjacent normal tissues (N1, N2, N3, N4 and N5)

A total of 27 815 circRNAs were detected in 5 pairs of HSCC tumorous and adjacent normal samples. Differential expression of circRNA in 5 paired samples could be visualized by using a scatter plot (Figure 1A). Furthermore, a volcano plot showed the differentially expressed circRNAs by the filtering criteria (FC ≥ 2.0, *P* value < .05) between cancerous samples and normal samples (Figure 1B). Consequently, we identified 155 differentially dysregulated circRNAs in HSCC tumour samples. Among them, 38 circRNAs were up‐regulated and 117 circRNAs were down‐regulated compared with the paired adjacent normal samples. Cluster heatmap clearly showed differentially dysregulated circRNAs between the cancerous samples and normal samples (Figure 1C), suggesting that circRNA expression between cancerous samples and normal samples were different.

### 3.2 CircMATR3 is abundantly expressed in HSCC tissues and mainly localized in the cytoplasm of FaDu cell

**Figure 2:**
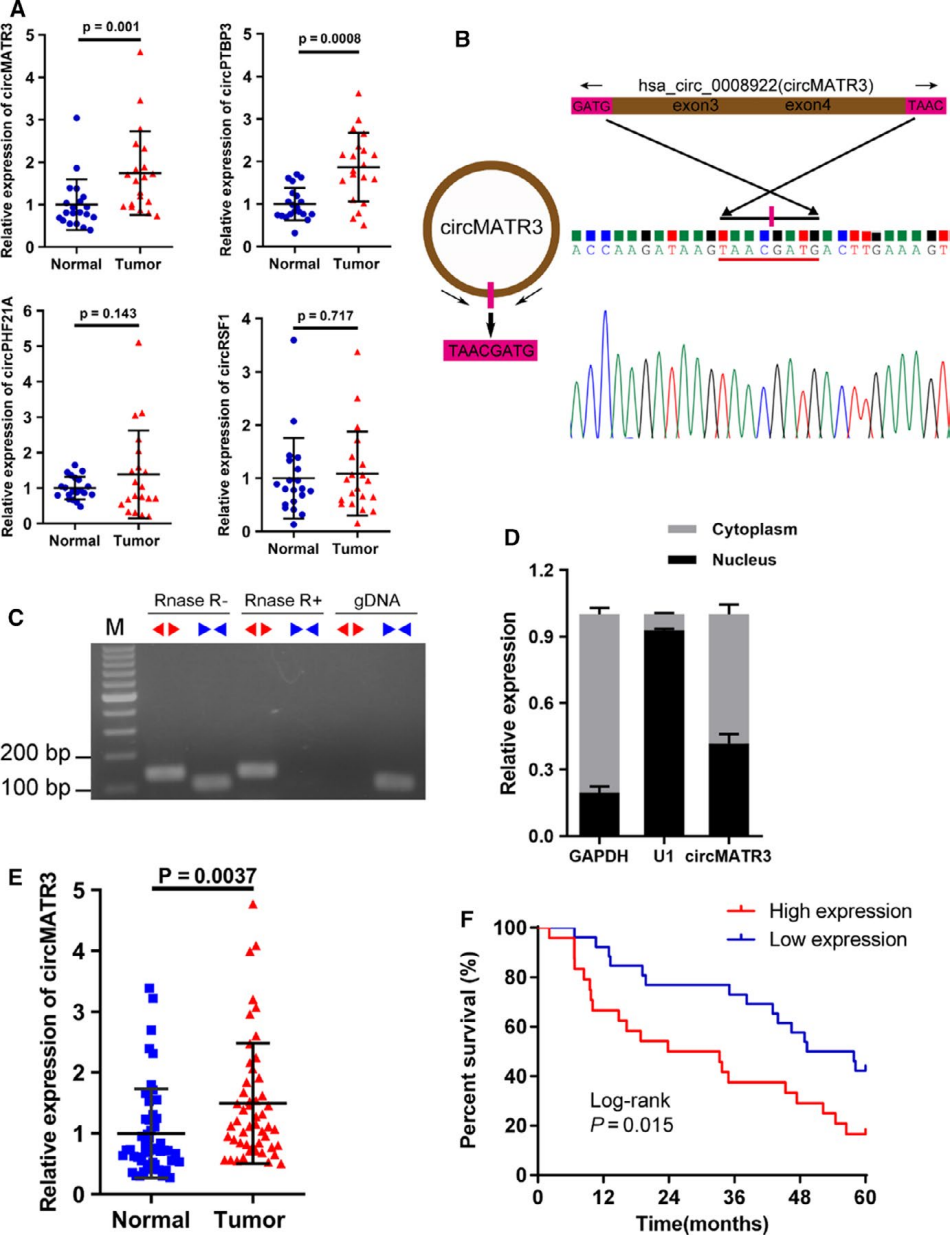
CircMATR3 overexpression in HSCC tissues and its association with prognosis of HSCC patients. A, Relative expression level of 4 corresponding circRNAs in 20 pairs of HSCC tissues and adjacent normal tissues measured by qRT‐PCR (n = 20, paired *t* test). B, The schematic illustration shows head‐to‐tail splicing site of circMATR3 PCR products amplified by divergent primers, and the spliced site is validated by Sanger sequencing. C, Total RNA with or without RNase R (4 U/mg) digestion were subjected to reverse transcription, and divergent primers and convergent primers were used to amplify the back‐spliced or linear products in cDNA and gDNA. The PCR products were examined via agarose gel electrophoresis. D, qRT‐PCR analysis indicates the distribution of GAPDH, U1 and circMATR3 in the nucleus and cytoplasm of HSCC cells (n = 5). E, The expression of circMATR3 was significantly increased in a total of 50 pairs of HSCC tissues and normal tissues (n = 50, paired *t* test). F, Kaplan‐Meier survival curves for 50 HSCC patients with low or high circMATR3 expression (Log‐rank test)

We verified the expression of these 4 (circMATR3, circPTBP3, circPHF21A and circRSF1) mostly dysregulated circRNAs in 20 pairs of HSCC tissues and their adjacent normal tissues by performing qRT‐PCR (Figure 2A). We found that the expression level of circMATR3 and circPTBP3 (generated from human gene PTBP3, termed circPTBP3, hsa_circ_0008192) was consistently with the result of circular RNA‐seq profiling, and the two circRNAs were markedly increased in HSCC tissues compared with that in paired tissues (Figure 2A). Although both circPTBP3 and circMATR3 expression was increased in HSCC tissues, the preliminary results exhibited that knock‐down of circPTBP3 did not affect the growth of HSCC cells (data not shown), whereas silencing of circMATR3 had a significant inhibitory effect on HSCC cell proliferation. Next, we confirmed the expression of circMART3 in another 30 pairs of HSCC tissues. Taken together, the results of qRT‐PCR showed that the expression level of circMATR3 was significantly increased in 50 pairs of HSCC tissues by comparing with that in the matched normal tissues (Figure 2E).

We then conducted Sanger sequencing to confirm the head‐to‐tail splicing junction in circMATR3 PCR product amplified with divergent primers (Figure 2B). In addition, we designed divergent primers for circMATR3 and convergent primers for MATR3 mRNA to amplify them. The PCR amplification products of circMATR3 were resistant to RNase R digestion, whereas the divergent primers amplified no products in genomic DNA (gDNA), which suggested that the result was not caused by PCR artefacts or genomic rearrangements (Figure 2C). In contrast, the PCR products of MATR3 mRNA amplified with convergent primers disappeared after digestion by RNase R (Figure 2C). By analysing the relative expression of cytoplasmic and nuclear circMART3 RNA, we also verified that circMATR3 predominantly localized in the cytoplasm of FaDu cells (Figure 2D). Taken together, these findings indicate that circMATR3 is abundant in HSCC tissues and stable in FaDu cells, which inspired us to investigate the significance of circMATR3 in HSCC pathogenesis.

### 3.3 Association of circMATR3 expression with clinicopathological characteristics and survival in HSCC patients

To further explore the association between the expression level of circMATR3 and clinicopathological features in HSCC patients, we separated the 50 patients into high‐ and low‐circMATR3 expression groups based on their median circMATR3 expression levels. As presented in Table 1, high circMATR3 expression level was significantly associated with late T classification (*P* = .022), advanced clinical stage (*P* = .026) and worse lymph node metastasis (*P* = .038). However, no significant associations were found between circMATR3 expression and other clinical characteristics such as sex, age, alcohol, smoking and tumour differentiation. Furthermore, we found that increased circMATR3 expression in HSCC tissues was markedly associated with poor survival for HSCC patients, as shown by the Kaplan‐Meier survival curve (Figure 2F). Taken together, these findings indicate that abundant circMATR3 expression may promote the progression and efficiently serve as a prognostic biomarker of HSCC.

**Table 1:** Relative circMATR3 expression and clinicopathological characteristics of 50 HSCC patients

Characteristics	Sum	CircMATR3 expression		*P* value
		Low	High	
Age (y)				.423
<60	18	8	10	
≥60	32	18	14	
Sex				.925
Male	45	24	21	
Female	5	2	3	
Smoking				.909
Never	7	3	4	
Ever	43	23	20	
Drinking				.546
Never	9	6	3	
Ever	41	20	21	
Differentiation				.272
Well or moderate	33	19	14	
Poor	17	7	10	
T category				.022
T1‐T2	23	16	7	
T3‐T4	27	10	17	
Lymph node metastasis				.038
Absent	20	14	6	
Present	30	12	18	
Clinical stage				.026
I‐II	16	12	4	
III‐IV	34	14	20	

Note

*P:* chi‐square test.

### 3.4 Influence of circMATR3 depletion on proliferation, cell cycle and apoptosis of FaDu cell

**Figure 3:**
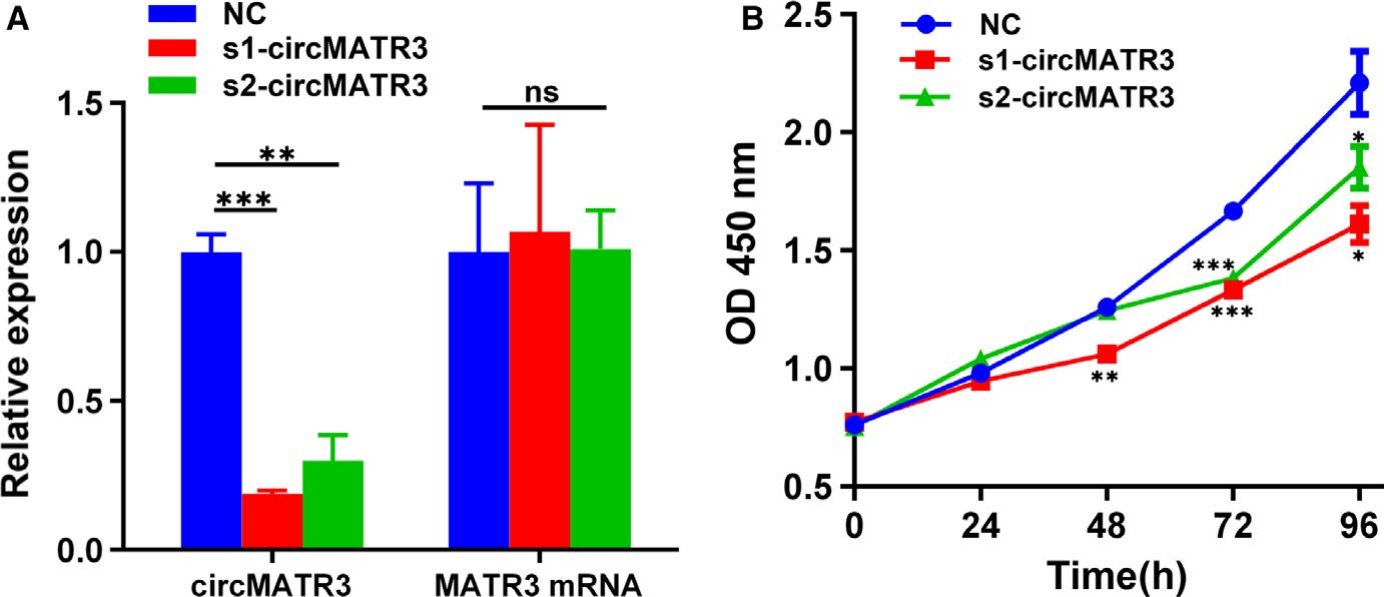
Inhibition of cell proliferation in FaDu cells by circMATR3 depletion. A, The silencing efficiency of circMATR3 and MATR3 mRNA was accessed by performing qPCR (n = 3). B, After transfected with NC, s1‐circMATR3 or s2‐circMATR3, cell proliferation was measured by CCK8 assays at indicated days (n = 3). *P < .05; **P < .01, ***P < .001

We conducted loss‐of‐function experiments to explore the biological function of circMATR3 in vitro. Compared with that in FaDu cells transfected with scrambled negative control (NC), the expression level of circMATR3 was significantly lower than in FaDu cells transfected with s1‐circMATR3 or s2‐circMATR3, while the expression level of MATR3 mRNA was not markedly changed (Figure 3A). The CCK8 assay demonstrated that knock‐down of circMATR3 significantly suppressed the proliferation of FaDu cells (Figure 3B), indicating that circMATR3 plays an important role in promoting the growth of FaDu cells.

**Figure 4:**
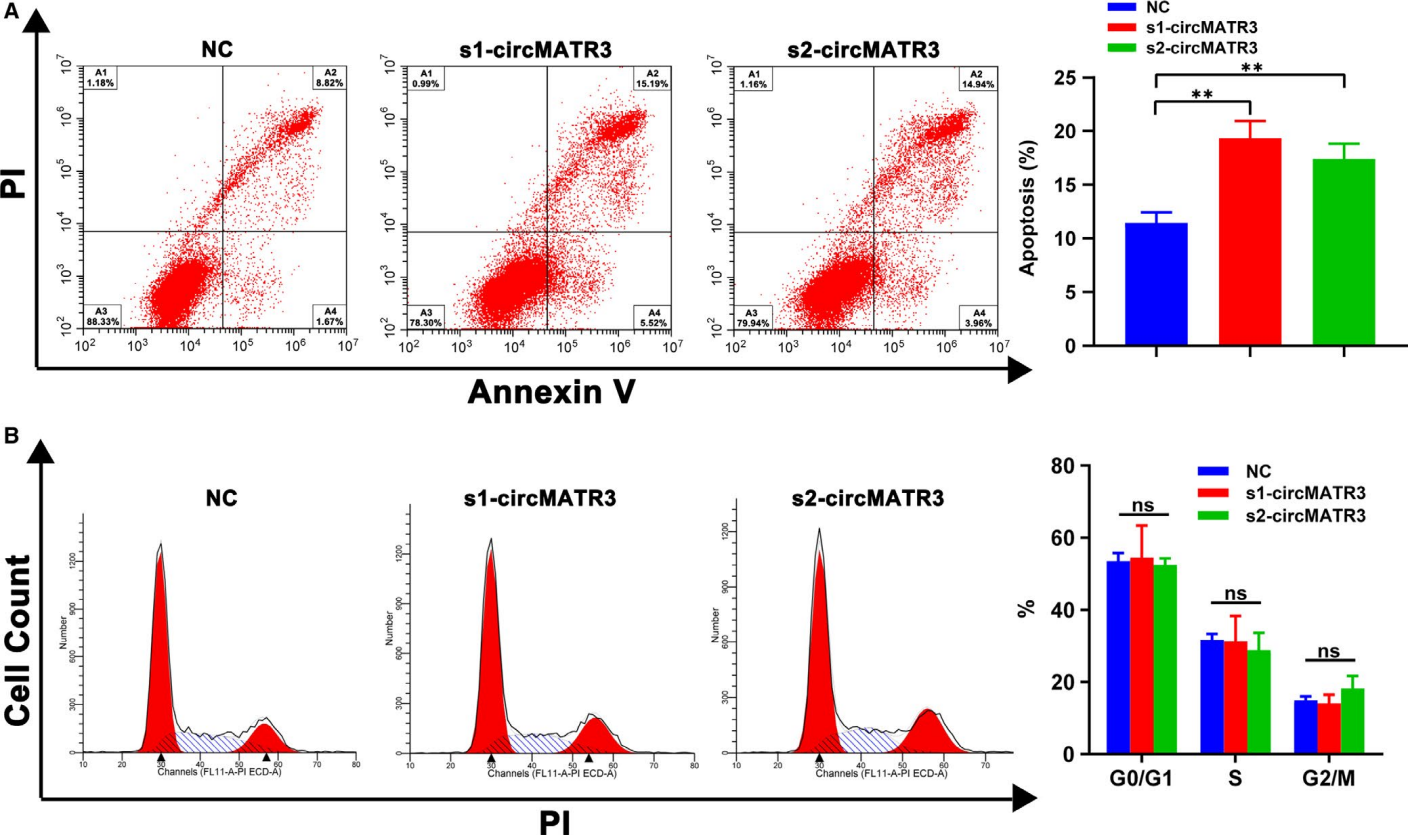
The effect of circMATR3 on hypopharyngeal cancer apoptosis and cell cycle in vitro. A, Effects of circMATR3 contribution to apoptosis of HSCC were analysed by using flow cytometry. Seventy‐two hours after transfection, the percentage of early apoptotic FaDu cells were accessed by conducting an annexin V‐FITC/PI dual staining assay (n = 3). **P < .01. B, Cell cycle of FaDu cells was determined by flow cytometry, which showed that knock‐down of circMATR3 did not affect cell cycle compared with negative control cells (n = 3)

Furthermore, flow cytometry was performed to explore whether circMATR3 promotes cell proliferation by influencing the cell cycle or apoptosis of FaDu cells. We observed that transfection with s1‐circMATR3 or s2‐circMATR3 induced apoptosis of FaDu cells (Figure 4A), suggesting that circMATR3 has anti‐apoptotic effect. However, the analysis of cell cycle via flow cytometry showed that there was no significant difference between FaDu cells transfected with s1‐circMATR3 or s2‐circMATR3 and those transfected with NC in any cell phase (Figure 4B).

Together, these results indicate that up‐expression of circMATR3 promotes the proliferation of HSCC through exerting anti‐apoptotic effect rather than accelerating cell cycle progression.

### 3.5 Contribution of circMATR3 to metastasis by increasing the invasion and migration of FaDu cells

**Figure 5:**
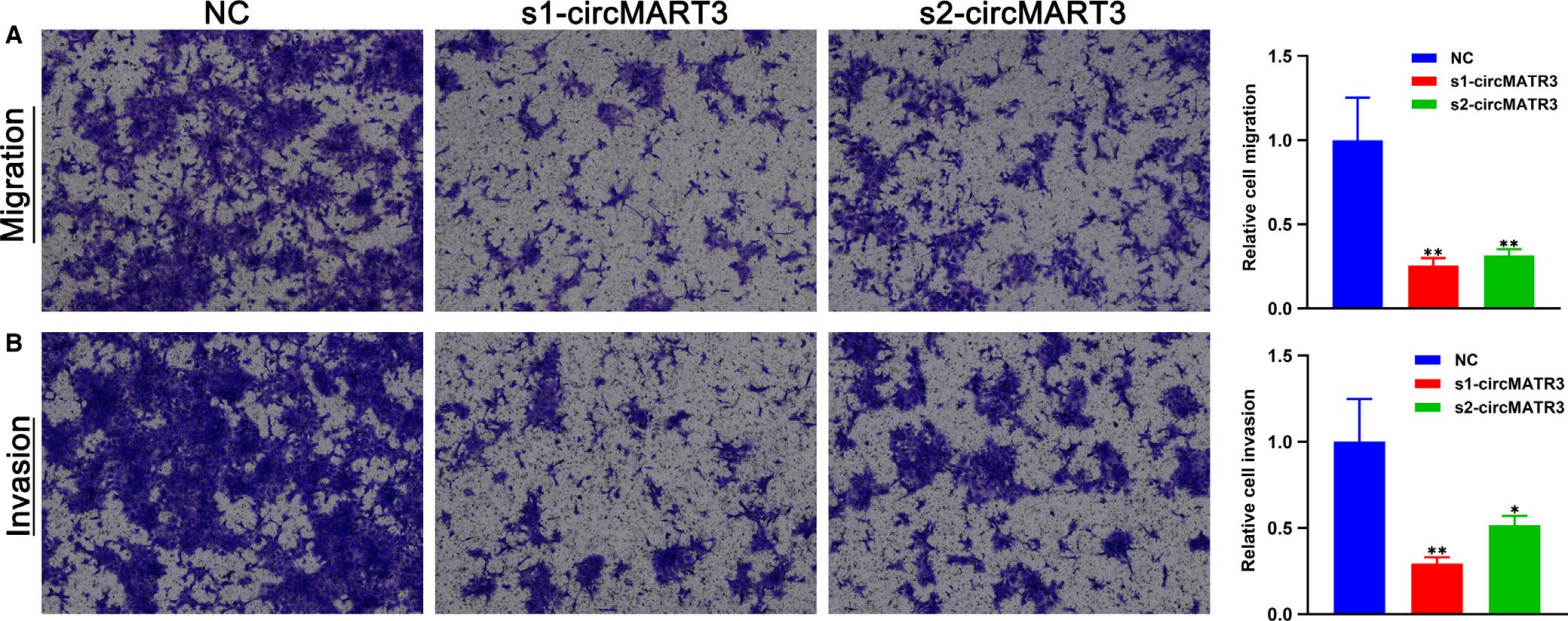
Silencing of circMATR3 weakened the migration and invasion of FaDu cells. FaDu cells were transfected with s1‐circMATR3 or s2‐circMATR3 or NC and cultured in corresponding chambers for 48 h. Migratory (A) and invasive (B) cells stained with crystal violet are presented (n = 3). *P < .05, **P < .01

To further understand the association between high expression level of circMATR3 and worse lymph node metastasis in HSCC patients, we performed transwell assays to determine whether circMATR3 promotes metastatic ability of HSCC cells. Silencing of circMATR3 in FaDu cells markedly decreased their migration (Figure 5A) and invasion (Figure 5B), indicating that circMATR3 could enhance the migration and invasion of HSCC cells. Therefore, we identified that circMATR3 also has a role in promoting metastasis in HSCC cells.

### 3.6 Prediction of circRNA‐miRNA‐mRNA networks and bioinformatics analysis for circMATR3

**Figure 6:**
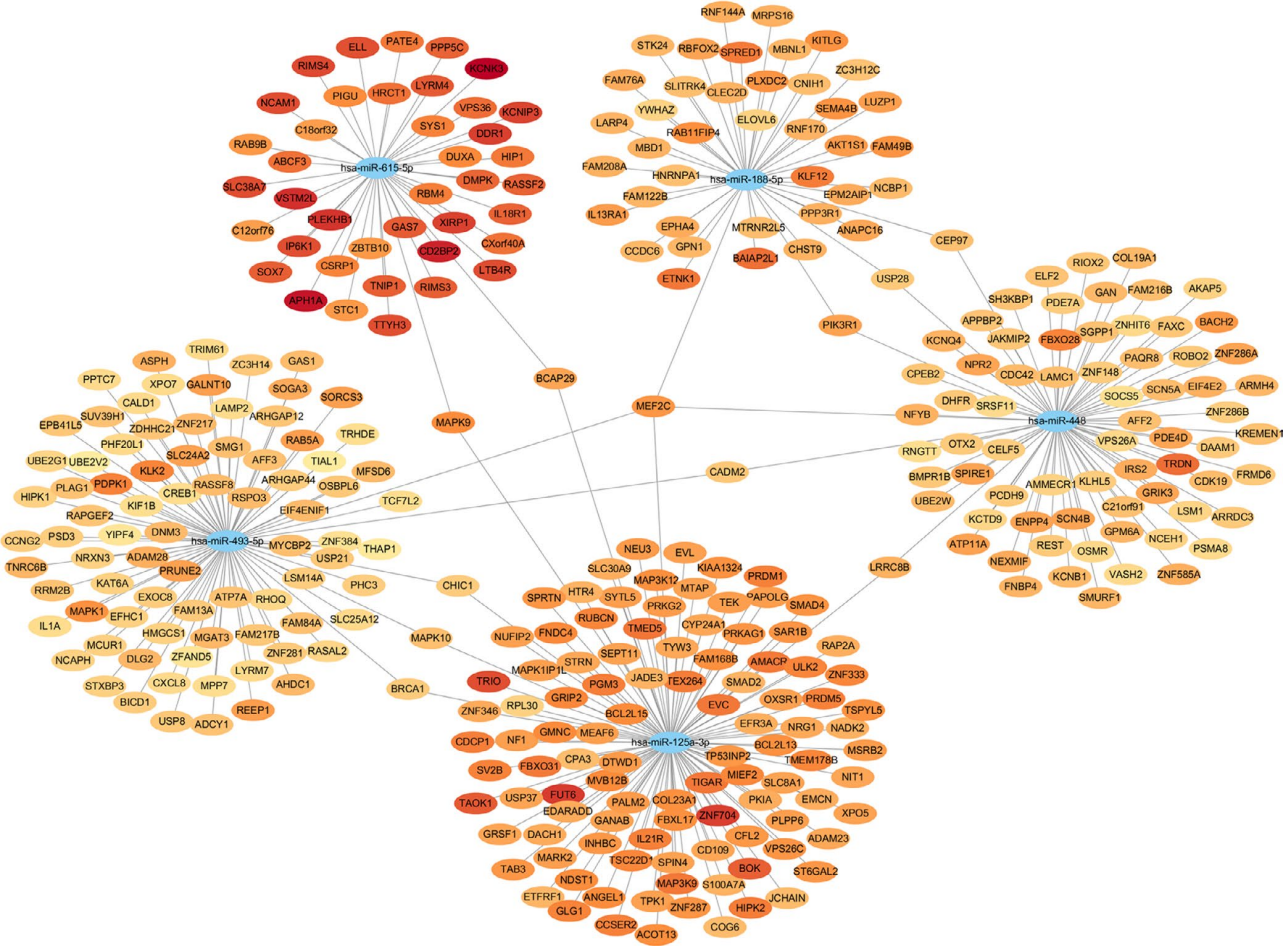
Bioinformatics prediction on circRNA‐miRNA‐mRNA network of circMATR3. The network diagram showing the target mRNAs of 5 highest‐ranking miRNAs matched circMATR3

In consideration of our results, it is highly likely that cicMATR3 is an extraordinary HSCC‐related circRNA. We hypothesized that circMATR3 might function as a miRNA sponge and regulate certain circRNA‐miRNA‐mRNA axes. Bioinformatics analysis suggested that circMATR3 could absorb multiple miRNAs. We then constructed circRNA‐ miRNA‐mRNA networks of 5 highest‐ranking miRNAs matched circMATR3 (Figure 6). Among hundreds of target mRNAs of these 5 miRNAs, 11 mRNAs were targeted by at least 2 miRNAs: MEF2C, BRCA1, MAPK10, CHIC1, MAPK9, BCAP29, PIK3R1, USP28, CEP97, LRRC8B and CADM2, which could be our key research focus in the future.

**Figure 7:**
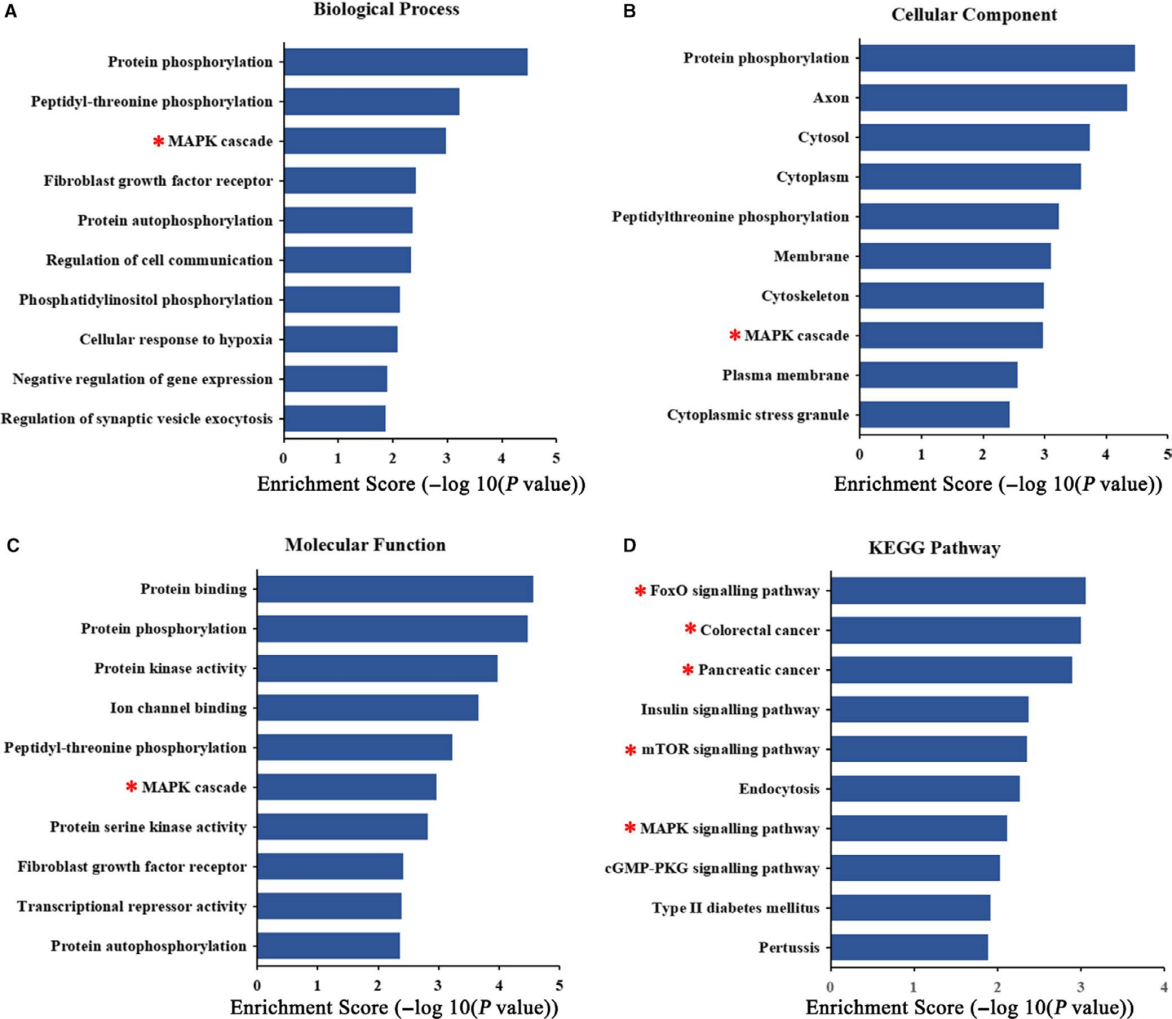
GO and KEGG pathway analysis on target mRNAs of 5 highest‐ranking miRNAs matched circMATR3. Top 10 markedly enriched biological process (A), cellular component (B), molecular function (C) and KEGG pathway (D) of the target mRNAs are functionally annotated by DAVID and KEGG pathway analysis

Moreover, GO and KEGG pathway analysis was performed to predict the functions of these target genes. The results indicated that these target genes of circMATR3 participated in various cellular processes, such as protein phosphorylation and MAPK cascade (Figure 7A‐C). Some vital cancer‐related pathways were shown to be affected by these target genes of circMATR3, including FoxO, mTOR and MAPK signalling pathways (Figure 7D), suggesting that circMATR3 might be a potential therapeutic target for HSCC.

## 4 DISCUSSION

Hypopharyngeal squamous cell carcinoma is one of the most malignant head and neck squamous cell carcinomas and is characterized by negligible symptoms in the early stages, early nodal metastasis and poor differentiation.^2^ Although great advancements have been made in the diagnosis and treatments for HSCC, the clinical outcomes of patients with HSCC are still far from satisfactory.^4^ Because early diagnostic biomarkers and new therapeutic targets of HSCC are lacking, it is imperative to improve our outstanding about molecular mechanisms in the carcinogenesis and progression of HSCC.

Recently, researchers found that more than 1000 circRNAs were stable in human serum exosomes and may serve as a potential biomarker for cancer detection,^22^, ^23^ and an increasing number of circRNAs have been reported to regulate the proliferation, apoptosis and metastasis in various human cancers. For instance, Li et al^24^ reported that circPVT1 was up‐regulated in non‐small cell lung cancer (NSCLC) specimens and that it could promote NSCLC cell proliferation and metastasis via sponging miR‐125b and activating E2F2 signalling pathway. However, the expression status, potential biological functions and molecular mechanisms of circRNAs in HSCC remain elusive.

In this study, we first utilized high‐throughput circRNA‐seq profiling to identify 155 differentially dysregulated circRNAs (FC ≥ 2.0, *P* value < .05), including 38 up‐regulated and 117 down‐regulated circRNAs in human HSCC samples. We for the first time characterized an abundant circRNA derived from exon3 and exon4 of MATR3 (termed circMATR3) and confirmed that the expression level of circMATR3 was markedly increased in HSCC tissues. In addition, our further analysis showed that increased expression of circMATR3 is significantly correlated with late T classification, advanced clinical stage, greater lymph node metastasis and poor survival, indicating that circMATR3 may be involved in the pathogenesis and prognosis of HSCC. Further in vitro experiments of HSCC cells demonstrated that circMATR3 promotes cell proliferation by suppressing apoptosis, and knock‐down of circMATR3 markedly inhibited invasion and migration of FaDu cells, suggesting that circMATR3 could function as an oncogenic circRNA in HSCC. These findings also partially explain the function of circMATR3 up‐expression in HSCC samples and its correlations with corresponding clinicopathological characteristics. Further investigation is needed to be made to identify the underlying molecular mechanistic details contributing to the oncogenic roles of circMATR3 in HSCC.

The molecular mechanism of most circRNAs is still not fully known.^11^ Recently, researches had proved that circRNAs could regulate gene expression by serving as cytoplasmic miRNA sponges and composing the circRNA‐miRNA‐mRNA network in many human cancers.^9^ The circRNA‐miRNA‐mRNA network is also known as competing endogenous RNA (ceRNA), which are considered as complicated post‐transcriptional regulatory axes mediated by miRNAs, and their deregulation could influence cancer development.^13^ Indeed, many circRNAs have been verified to participate in the development and progression of various cancers by serving as miRNA sponges. For instance, circ‐Sry contains 16 binding sites of miR‐138 and interacts with miR‐138.^9^ The CDR1as/ciRS‐7 harbours more than 70 binding sites of miR‐7 and could sequester miR‐7 to reduce its tumour‐suppressive roles in colorectal cancer and nasopharyngeal carcinoma.^9^, ^25^, ^26^ CircHIPK3 was proved to be a tumour‐related molecule in bladder cancer by sequestering miR‐558.^27^ CircMTO1 serves as the sponge of miR‐9 to suppress the progression of hepatocellular carcinoma,^28^ and circ‐ITCH suppresses the wnt/β‐catenin pathway by acting as a sponge of multiple miRNAs (miR‐7, miR‐20a and miR‐214).^29^ Consistent with our findings, we verified that circMATR3 is predominantly located in the cytoplasm of FaDu cells, which suggested that sponging particular miRNAs might be the potential molecular mechanism by which circMATR3 participates in the proliferation, metastasis and apoptosis of HSCC cells.

Therefore, with bioinformatics analysis, we predicted that circMATR3 could sponge multiple miRNAs. We selected five highest‐ranking miRNAs matched circMATR3 and performed GO and KEGG pathway analysis for their target genes. Among these 5 miRNAs, we found that miR‐188‐5p and miR‐448 are important tumour‐suppressive molecules. Fang et al^30^ reported that miR‐188‐5p suppresses tumour cell proliferation and metastasis by directly targeting FGF5 in hepatocellular carcinoma, implying that up‐regulated circMATR3 might sponge miR‐188‐5p and abolish its inhibitory effects on cell proliferation and metastasis. Similarly, Wu et al^31^ showed that elevated expression of miR‐448 suppressed osteosarcoma cell proliferation and invasion through targeting EPHA7, indicating that circMATR3 may reverse the inhibitory roles by absorbing miR‐448. Interestingly, miR‐188‐5p and miR‐448 have 3 common targeted mRNAs; among them, USP28 is a famous oncogene that is required for MYC stability in several human tumour cells,^32^ while MYC is a central regulator of cell growth, proliferation and apoptosis in many human cancers.^33^ In the future, we will perform RIP, RNA pull‐down assays and luciferase reporter assays to verify the regulatory axes.

Furthermore, we demonstrated that circMATR3 is an oncogenic circRNA that could promote the metastasis of HSCC cells, which is similar with others' findings. Although many circRNAs have been reported to enhance metastasis of multiple human cancers, to our knowledge, no studies have verified the function of circMATR3 in HSCC, let alone its downstream target and pathway in HSCC. Thus, we performed GO and KEGG pathway analysis, and the results indicated that circMATR3 may exert its oncogenic role by serving as miRNA sponges and regulating several signalling pathways. Notably, the results also revealed that MAPK cascades could be predicted in three aspects of GO enrichment analysis and KEGG pathway analysis. As known to all, MAPK cascades are central signalling pathways that could regulate various stimulated cellular processes, including cell proliferation, apoptosis and metastasis.^34^ Similarly, protein phosphorylation is enriched mostly in both biological process and molecular function of the GO result, and phosphorylation is one of the most extensively post‐translational modifications, which regulates many cellular activities, such as cell growth, apoptosis and cell signalling in healthy condition. The alterations in protein phosphorylation pathways could lead to cancer development. The signalling pathway like MAP kinase plays a major role in the cell growth, and deregulation in its phosphorylation‐dephosphorylation cascade has been manifested in various types of cancers.^35^ Therefore, we assumed that MAPK cascade and protein phosphorylation might be potential signalling pathways, in which circMATR3 may play an oncogenic role in growth and progression of HSCC. Nevertheless, these hypotheses should be further validated in our future studies.

There are several limitations in our study. First, in vitro experiments of this study were only performed in FaDu cells; however, currently we can obtain only one HSCC cell line in China, whereas it would be more appropriate if the role of circMATR3 were conducted in more than one HSCC cell line. Additionally, to confirm the roles of circMATR3, in vivo experiments should be further conducted in the mouse models we have established before.^36^ Therefore, the roles of circMATR3 in HSCC warrant further investigation in our future studies.

## CONFLICT OF INTEREST

The authors confirm that there are no conflicts of interest.

## AUTHOR CONTRIBUTIONS

ZW, PW, DW, SC, HL, LC, XZ and CL carried out the majority of the experiment, data analysis and wrote the manuscript. They were all helped by GL, JY, XP and DL. All authors reviewed and approved the final manuscript.

## Supporting information

Table S1

## Data Availability

The data that support the findings of this study are available on request from the corresponding author. The data are not publicly available due to privacy or ethical restrictions.
