# Supplementary material for: Effect of up‐regulation of circMATR3 on the proliferation, metastasis, progression and survival of hypopharyngeal carcinoma
Source: J Cell Mol Med. 2020 Mar 12;24(8):4687–97. doi: 10.1111/jcmm.15134 (PMC7176838; doi:10.1111/jcmm.15134)
Supplement: Supplementary file 1 — Table S1 [file JCMM-24-4687-s001.docx]

**Supplementary Table S1:** The sequences of primers and siRNA used in this study

| **The sequences of primers for PCR (5′→3′)** | |
| --- | --- |
| MATR3 mRNA | Forward: CTACACGGGAGCCACCATAC |
| MATR3 mRNA | Reverse: GGGTTGAGACTAGGACCACG |
| circMATR3 | Forward: TCACCTGAATGACATCTACCTCC |
| circMATR3 | Reverse: GTCACCTGCCACTATTTCCTCC |
| circPTBP3 | Forward: TGTCAGTGCCGTCCAATCAGG |
| circPTBP3 | Reverse: TGCCAGAAGAAAGAAGCTCATCAG |
| circPHF21A | Forward: ACAGGAGGCTCTTAAAGTGGAAA |
| circPHF21A | Reverse: ACTGGCTCTCCTTCAGCTCTC |
| circRSF1 | Forward: TCATTCCTCCACAAGAGCCAGAC |
| circRSF1 | Reverse: ATCGCAAGAGTCACACAGAAGAA |
| 18s rRNA | Forward: TTAATTCCGATAACGAACGAGA |
| 18s rRNA | Reverse: CGCTGAGCCAGTCAGTGTAG |
| GAPDH | Forward: GCACCGTCAAGGCTGAGAAC |
| GAPDH | Reverse: TGGTGAAGACGCCAGTGGA |
| U1 | Forward: TCCCAGGGCGAGGCTTATCCATT |
| U1 | Reverse: GAACGCAGTCCCCCACTACCACAAAT |
| **The sequences of siRNAs (5′→3′)** | |
| NC | Sense: UUCUCCGAACGUGUCACGU |
| NC | Antisense: ACGUGACACGUUCGGAGAA |
| s1-circMATR3 | Sense: AAGAUAAGUAACGAUGACU |
| s1-circMATR3  s2-circMATR3  s2-circMATR3 | Antisense: AGUCAUCGUUACUUAUCUU  Sense: UAACGAUGACUUGAAAGUA  Antisense: UACUUUCAAGUCAUCGUUA |
